# Adiponectin and chronic kidney disease; a review on recent findings

**Published:** 2015-07-27

**Authors:** Maryam Heidari, Parto Nasri, Hamid Nasri

**Affiliations:** ^1^Department of Internal Medicine, Division of Nephrology, Shahrekord University of Medical Sciences, Shahrekord, Iran; ^2^Department of Nephrology, Isfahan University of Medical Sciences, Isfahan, Iran

**Keywords:** Adeponectin, Chronic kidney disease, Hemodialysis

## Abstract

Adiponectin is a multifunctional cytokine that has a role in regulating inflammation. In patients without chronic renal failure (CRF) and type 2 diabetics, decreased adiponectin levels are associated with insulin resistance. Lower serum plasma adiponectin values are link to larger tumor size and metastasis in clear-cell carcinoma of the kidney too. However, in patients with established chronic kidney disease (CKD), adiponectin levels are elevated and positively predict progression of disease. In addition, increased levels of serum adiponectin of hemodialysis patients were associated with decrease in bone mineral density in hemodialysis patients. Thus, depending on type of renal failure should be adjusted the adiponectin levels in patients. In CKD patients without diabetic, decreasing adiponectin levels by ARB drugs may be appropriate for inhibition of disease progression.

**Implication for health policy/practice/research/medical education:**

Adiponectin is a multifunctional cytokine that has a role in regulating inflammation. In patients without chronic renal failure (CRF) and type 2 diabetics, decreased adiponectin levels are associated with insulin resistance. However, in patients with established chronic kidney disease, adiponectin levels are elevated and positively predict progression of disease.

## Introduction

Evidences are accumulating that adipose tissue releases various active metabolic compounds, including pro-inflammatory cytokines (1). These compounds consist, irisin, leptin, adipsin, resistin, angiotensinogen, tumor necrosis factor-a (TNF-a), plasminogen activator inhibitor type-1 and interleukin (IL6). Therefore, patients whose clearance of cytokines is impaired, as in chronic kidney disease (CKD), may be prone to insulin resistance and accelerated atherosclerosis (2).

Adiponectin is a 244 amino acid, 30 kDa protein encoded by the apM1 gene and related to a group of adipocyte-specific secretory proteins known as “adipokines” (3). Adiponectin circulates in human plasma in three major oligomeric forms: a low-molecular weight (LMW) trimer, a middle-molecular weight (MMW) hexamer, and high-molecular weight (HMW) 12- to 18-mers (4). Adiponectin is reported to be rich in human blood, with its plasma levels in the mg/ml range and, thus, accounting for 0.01% of total plasma protein (5). Adiponectin is an adipokine with anti-atherogenic properties (6).

High circulating adiponectin is strongly associated with reduced cardiovascular risk and low adipose tissue adiponectin transcriptional expression is associated with hypoadiponectinemia and could influence the insulin resistance and cardiovascular disease in obesity and type 2 diabetes (7). Chronic kidney disease (CKD) is a unique condition with exceedingly high incidence of insulin resistance and cardiovascular morbidity and mortality (8,9), and is paradoxically associated with elevated plasma adiponectin.

Plasma adiponectin level is dependent on kidney function, being markedly in­creased among patients with kidney impairment (10).

Therefore, in this review article, we performed a Medline (PubMed) search, to identify articles published during the last 10 years (with special focus on papers published during the last 3 years), changing the adiponectin levels in CKD patients under clinical conditions.

## Clinical implications

Ho et al (11) reported that uremic patients had one-third of the levels of adiponectin (*P*<0.001) in compared with nonuremic patients, while females had a 1.5-fold higher levels of adiponectin.

Plasma adiponectin level was significantly higher in CKD patients than control group (*P*<0.05) and in CKD patients plasma adiponectin inversely correlated with glomerular filtration rate (GFR) (r=-0.570, *P*<0.001) (12) and creatinine (r=-0.423; *P*<0.01) (13). Another study showed that adiponectin correlated inversely with GFR (r=-0.45; *P*<0.001), body mass index (BMI) (r=-0.33; *P*<0.01), and visceral fat (r=-0.49; *P*<0.001), while a positive association with amount of proteinuria was detected (r=0.21; *P*<0.05) (14).

The mean adiponectin levels in the pre-dialysis groups were significantly lower than in the chronic ambulatory peritoneal dialysis (CAPD) group (*P*<0.05). The levels of adiponectin were increased for all patients with chronic renal failure (CRF) (15).

For patients with normal range GFR and type 2 diabetes, albuminuria inversely correlated with plasma adiponectin (r=−0.31, *P*<0.05) (16), while plasma adiponectin positively correlates to intercellular leukocyte adhesion molecule (17). Therefore, adiponectin is associated with parameters of kidney function at the stage of apparently normal kidney function in type 2 diabetes (16).

Lenghel et al (17) concluded that median adiponectin was not significantly different in diabetic and non-diabetic subjects, however, the odds ratio comparing the highest tertile to the lower two tertiles was significant (1.9; 95% CI, 1.1, 3.6). In addition, higher adiponectin was independently associated with lower eGFR and higher urinary albumin levels (17).

Markedly elevated plasma adiponectin serum values were observed in CKD patients (18,19). In hemodialysis patients, the plasma levels of HMW adiponectin were significantly higher than CKD patients (20). In hemodialysis patients, adiponectin is an indicator for high-density lipoprotein cholesterol (HDL-C) and total-cholesterol levels and is twice its values in these patients (21).

Multivariate analysis for kidney transplant recipient patients showed that the presence of metabolic syndrome early after transplantation was independently associated with decreased plasma values of adiponectin (β: -6.39, r (2) 0.195, *P*<0.0001) and increased risk for clinical events (OR: 5.6, 95% CI: 1.9, 16.5; *P*<0.01) (22). Taherimahmoudi et al (23), described that adiponectin levels were remarkably higher in the patient group before transplantation when compared with healthy subjects (*P*<0.001) and after transplantation this level remained significantly higher (*P*<0.001). In post-transplant south Asian patients the level of total and HMW adiponectin are lower and may be a novel marker for cardiovascular risk factor (24).

Okuno et al (25), demonstrated that in hemodialysis patients increased levels of serum adiponectin were related with decreases in bone mineral density. Therefore adiponectin may has participation in bone resorption especially in end-stage renal disease (ESRD) patients (25). Other changes in adiponectin level include decreased level in clear-cell carcinoma of the kidney depend on tumor size and metastasis (26), and in carotid arteriosclerosis (27). However, there is no significant relationship between adiponectin level and all-cause mortality and cardiovascular death (28). Zoccali et al (29), emphasized that low plasma level of adiponectin and high level of norepinephrine contributing to reverse cardiovascular events in nondiabetic hemodialysis (HD) patients.

In patients with CKD changes in HMW-adiponectin was significantly correlated with changes in eGFR (r=0.597, *P*=0.001) (30). In another investigation, although 60% of subjects with CKD have CAD, plasma levels of adiponectin were not decreased in subjects with CRF compared with controls (17.02±9.8 versus 16.40±9.0; *P*=0.78). Urinary adiponectin levels, associates inversely with GFR (r=−0.4; *P*<0.05) and plasma adiponectin levels (r=0.9; *P*<0.001) (31). In hypertensive stage III-IV CKD individuals, no alterations in inflammatory markers, total or HMW adiponectin was detected (32). In individuals with CAD, metabolic syndrome is related to a lower serum HMW adiponectin, while the presence of CKD is associated with increasing of the serum HMW adiponectin (33). In obesity, CKD at early stages develops in parallel with atherosclerotic process of the carotid arteries, which correlates with attenuation of organ-protecting properties of adiponectin (34). To found the effect of losartan of the level of adiponectin, we recently conducted a randomized double blind clinical trial investigation, on a group of non-diabetic individuals, who were on routine hemodialysis program. In our study, exclusion criteria were presence of chronic active or infections, taking angiotensin converting enzyme or renin–angiotensin system blockers or presence of diabetes. Individuals were allocated into two groups. First group was received losartan 12.5 mg twice in a day for the first week, then 25 mg twice/day during the second week and finally, they received 75 mg/day (50 mg in morning, 25 mg for evening) from the third week to the end of 16thweek. Hemodialysis subjects of the second group received placebo. Our patients consisted of 73 non-diabetic hemodialysis participants (females=33) enrolled to the study. The range of subjects’ age was from 13 to 91 years. In our investigation, the mean (±SD) of serum adiponectin value in all subjects was 10.6 (±3.9) µg/ml. In our study, a significant reduction of serum adiponectin serum value after 4 months of treatment by losartan (8.86±3.43 of interventional group versus 10.71±3.94 control group; *P*<0.05) was detected. Interestingly, none of the patients had serum potassium value >5 mg/dl or episode of hypotension during our study. In this study, we concluded that, the diminution in serum adiponectin value in non-diabetic patients on routine hemodialysis by losartan might offer potential protection in these groups of patients. However, the mechanism liable for this reduction remains to be investigated (35).

**Table 1 در متنرفرنس ندارد:** The clinical impact of adiponectin in CKD patients

**Study**	**Population**	**Finding**
Sedighi and Abediankenari (12)	42 CKD patients and 46 healthy persons	Plasma adiponectin level was significantly higher in CKD patients than control group (*P*<0.05) and inversely correlated with GFR (r=-0.570, *P*<0.001).
Kir et al (15)	37 patients with CKD on conservative treatment, 34 PD on CAPD, 35 HD and CAPD, and 25 healthy volunteers	The mean adiponectin levels in the predialysis groups were significantly lower than in the CAPD group (*P*<0.05). The levels of adiponectin was increased for all patients with CRF.
Stępień et al (13)	67 non-diabetic obese: patients without chronic CKD (n=52)	Negative correlations occurred between creatinine and visceral adiposity index (r=-0.332; *P*<0.05), body adiposity index (r=-0.619; *P*<0.0001), and adiponectin (r=-0.423; *P*<0.01).
Lenghel et al (17)	79 consecutive type 2 diabetic outpatients and 46 controls	Plasma adiponectin positively correlates to intercellular leukocyte adhesion molecule.
Barlovic et al (16)	52 patients with normal range GFR and type 2 diabetes	Albuminuria correlated with plasma adiponectin (r=-0.31, *P*<0.05). Adiponectin is associated with parameters of kidney function already at the stage of apparently normal kidney function in type 2 diabetes.
Ho et al (11)	71 patients	Compared with nonuremic patients, uremic patients had one-third of the levels of adiponectin (*P*<0.001). Females had 1.5-fold higher levels of adiponectin.
Mills et al (36)	201 patients with CKD and 201 controls without	Median adiponectin was not significantly different in cases and controls, but the odds ratio comparing the highest tertile to the lower two tertiles was significant (1.9; 95% CI, 1.1, 3.6). In addition, higher adiponectin was independently associated with lower eGFR and higher urinary albumin levels.
Kamimura et al. (14)	98 CKD patients	Adiponectin correlated with GFR (r=-0.45; *P*<0.001), proteinuria (r=0.21; *P*<0.05), BMI (r = -0.33; *P*<0.01), and visceral fat (r=-0.49; *P*<0.001).
Jorsal et al (37)	58 with normoalbuminuria, 43 with persistent microalbuminuria, and 44 with persistent macroalbuminuria	Urinary adiponectin increased with increasing levels of urinary albumin excretion (*P*< 0.01). Urinary adiponectin was associated with markers of tubular damage (*P*<0.01).
Shoji et al (38)	103 patients with ESRD undergoing HD and 166 healthy subjects	Plasma adiponectin correlated negatively with plasma TG and positively with HDL-C in both healthy and ESRD groups.
Elshamaa et al (18)	78 advanced CKD (stages 4 and 5) pediatric patients undergoing maintenance HD or CT	Markedly (*P*<0.01) elevated plasma adiponectin levels were detected in CKD patients, especially CT patients, compared to control subjects.
Nakagawa et al (20)	144 HD patients and 30 patients with CKD	Plasma HMW adiponectin levels in hemodialysis patients were significantly higher than those in patients with CKD, negatively associated with visceral fat area and serum TG and positively associated with plasma total adiponectin.
Ribeiro et al (21)	187 HD patients and 25 healthy	Adiponectin almost doubled its values in patients and seems to be an important determinant in HDL-C and total cholesterol levels, improving the lipid profile in these patients.
Roubicek et al (19)	15 women with ESRD and 17 healthy women	Serum concentrations of adiponectin was significantly higher in the ESRD versus control group.
Kaynar et al (39)	150 patients, without active infections or chronic inflammatory conditions	Adiponectin and resistin levels in predialysis, peritoneal dialysis and hemodialysis patients were significantly higher than control group (*P*<0.001). This study had given significant positive correlations between presence of PEW and serum adiponectin levels (r=0.349, *P*< 0.001). High serum resistin and adiponectin levels might have a role in development of PEW among dialysis patients.
Campbell et al (32)	20 hypertensive stage III-IV CKD patients	There was no change in inflammatory markers, total or HMW adiponectin.
Landau et al (40)	2418 individuals without reported diabetes at baseline	Adiponectin was associated with IR in those without CKD but not in those with CKD. In mainly Stage 3 CKD, kidney function is associated with IR; except for adiponectin, the correlates of IR are similar in those with and without CKD.
Fonseca et al (41)	40 consecutive adult patients with ESRD who were undergoing kidney transplantation	Kidney graft function is an independent determinant of leptin levels, but not of adiponectin.
Alam et al (42)	987 prevalent KTR on all-cause mortality and death-censored graft failure	Elevated adiponectin levels are associated with higher risk for death but not allograft failure in prevalent KTR.
Kulshrestha et al (22)	74 previously nondiabetic KTR patients	Multivariate analysis showed that the presence of metabolic syndrome early after transplantation was independently associated with depressed plasma adiponectin levels (β -6.39, r(2) 0.195, *P*<0.0001) and increased risk for clinical events (OR: 5.6, 95% CI: 1.9, 16.5; *P*<0.01).
Taherimahmoudi et al (23)	67 candidates with ESRD along with 30 healthy unrelated donors	Adiponectin levels were remarkably higher in the patient group before transplantation when compared with healthy subjects (*P*<0.001) and remained significantly higher thereafter (*P*<0.001).
Prasad et al (24)	129 clinically stable age-matched KTR	Total and HMW adiponectin concentrations are lower in KTR and may be promising exploratory biomarkers of post-transplant cardiovascular risk.
Okuno et al (25)	114 male HD patients	Increased levels of serum adiponectin were associated with decrease in bone mineral density in male hemodialysis patients. Adiponectin may play a role in mineral and bone disorder, possibly in bone resorption, of patients with CKD 5D.
Wang et al (28)	238 ESRD patients on maintenance PD	Plasma adiponectin showed no significant association with all-cause mortality and cardiovascular death.
Hayashi et al (27)	95 CKD patients without dialysis and 81 non-CKD patients	Higher adiponectin levels were observed in CKD patients compared with non-CKD patients. After adjusting for other risk factors, low levels of adiponectin were independently correlated with carotid arteriosclerosis in CKD patients
Toyama et al (30)	Patients with CAD	Changes in HMW-adiponectin was significantly correlated with changes in eGFR (r= 0.597, *P*<0.001).
Yaturu et al (31)	43 subjects with CKD compared with those of 34 control subjects	Although 60% of subjects with CKD have CAD, plasma levels of adiponectin were not decreased in subjects with CKD compared with controls (17.02 ± 9.8 vs. 16.40 ± 9.0 with *P*=0.78). Urinary adiponectin levels correlate inversely with GFR (r=−0.4; *P*<0.05) and plasma adiponectin levels (r = 0.9; *P*<0.0001).
Hara et al (33)	228 consecutive patients with CAD	In individuals with CAD, metabolic syndrome is associated with a lower serum HMW adiponectin, while the presence of CKD is associated with elevation of the serum HMW adiponectin.
Saginova et al (34)	86 obese patients	In obesity, CKD at early stages develops in parallel with atherosclerotic lesion of the carotid arteries, which correlates with attenuation of organ-protecting properties of adiponectin.
Zoccali et al (29)	192 nondiabetic HD patients	Low adiponectin and high norepinephrine seem to be interacting factors in the dismal cardiovascular outcomes with ESRD.
Pinthus et al (26)	42 patients with clear-cell RCC, including 15 with metastatic disease	Lower plasma adiponectin levels are associated with larger tumor size and metastasis in clear-cell carcinoma of the kidney.
Mardani et al (35)	73 non-diabetic HD patients	A significant decrease of serum adiponectin level after four months of treatment by losartan (8.86±3.43 of interventional group versus 10.71±3.94 control group; *P*<0.05) was observed..

Abbreviations‏: CKD, chronic kidney disease; HD, hemodialysis; PD, peritoneal dialysis; GFR, glomerular filtration rate; CAD, chronic kidney disease; CAPD, continuous ambulatory peritoneal dialysis; ESRD, end-stage renal disease; HMW, high-molecular weight; HDL-C, high-density lipoprotein cholesterol; TG, triglycerides; KTR, kidney transplant recipients; PEW, protein-energy wasting; BMI, body mass index; CT, conservative treatment; IR, insulin resistance; OR, odds ratio; RCC, renal cell carcinoma.

## Conclusion

Adiponectin is a multifunctional cytokine which has a role in regulating inflammation. In patients without CKD such as type 2 diabetics, decreased adiponectin levels are associated with insulin resistance. Lower plasma adiponectin levels are associated with larger tumor size and metastasis in clear-cell carcinoma of the kidney. However, in patients with established CKD, adiponectin levels are elevated and positively predict progression of disease. In addition, increased levels of serum adiponectin of hemodialysis patients were associated with decrease in bone mineral density in hemodialysis patients. Thus, depending on type of renal failure should be adjusted the adiponectin levels in patients. In CKD patients without diabetic, decreasing adiponectin levels by angiotensin II receptor blockers (ARBs) may be appropriate for inhibition of disease progression.

## Authors’ contribution

PN and MH reviewed the literatures and wrote the manuscript. HN edited the paper.

## Conflicts of interest

The authors declared no competing interests.

## Ethical considerations

Ethical issues (including plagiarism, misconduct, data fabrication, falsification, double publication or submission, redundancy) have been completely observed by the authors.

## Funding/Support

None.
